# Microscopy Image Dataset for Deep Learning-Based Quantitative Assessment of Pulmonary Vascular Changes

**DOI:** 10.1038/s41597-024-03473-z

**Published:** 2024-06-15

**Authors:** Aleksandr M. Sinitca, Asya I. Lyanova, Dmitrii I. Kaplun, Hassan Hassan, Alexander S. Krasichkov, Kseniia E. Sanarova, Leonid A. Shilenko, Elizaveta E. Sidorova, Anna A. Akhmetova, Dariya D. Vaulina, Andrei A. Karpov

**Affiliations:** 1 Centre for Digital Telecommunication Technologies, St. Petersburg Electrotechnical University “LETI”, St. Petersburg, 197022 Russia; 2 Artificial Intelligence Research Institute, China University of Mining and Technology, Xuzhou, 221116 China; 3 Department of Automation and Control Processes, St. Petersburg Electrotechnical University “LETI”, St. Petersburg, 197022 Russia; 4 Radio Engineering Systems Department, St. Petersburg Electrotechnical University “LETI”, St. Petersburg, 197022 Russia; 5 Department of Computer Science and Engineering, St. Petersburg Electrotechnical University “LETI”, 197022 Saint Petersburg, Russia; 6 Institute of Experimental Medicine, Almazov National Medical Research Centre, St. Petersburg, 197341 Russia

**Keywords:** Hypertension, Molecular imaging

## Abstract

Pulmonary hypertension (PH) is a syndrome complex that accompanies a number of diseases of different etiologies, associated with basic mechanisms of structural and functional changes of the pulmonary circulation vessels and revealed pressure increasing in the pulmonary artery. The structural changes in the pulmonary circulation vessels are the main limiting factor determining the prognosis of patients with PH. Thickening and irreversible deposition of collagen in the pulmonary artery branches walls leads to rapid disease progression and a therapy effectiveness decreasing. In this regard, histological examination of the pulmonary circulation vessels is critical both in preclinical studies and clinical practice. However, measurements of quantitative parameters such as the average vessel outer diameter, the vessel walls area, and the hypertrophy index claimed significant time investment and the requirement for specialist training to analyze micrographs. A dataset of pulmonary circulation vessels for pathology assessment using semantic segmentation techniques based on deep-learning is presented in this work. 609 original microphotographs of vessels, numerical data from experts’ measurements, and microphotographs with outlines of these measurements for each of the vessels are presented. Furthermore, here we cite an example of a deep learning pipeline using the U-Net semantic segmentation model to extract vascular regions. The presented database will be useful for the development of new software solutions for the analysis of histological micrograph.

## Background & Summary

Pulmonary hypertension is a **syndrome** that accompany a number of diseases of different etiologies, united by basic mechanisms of structural and functional changes of the pulmonary circulation vessels and presented by increased pressure in the pulmonary artery. The prevalence of pulmonary hypertension, regardless of etiology, is about 1 percent of the global population^1^. A special place among the various forms of pulmonary hypertension is occupied by idiopathic pulmonary arterial hypertension (iPAH) and chronic thromboembolic pulmonary hypertension (CTEPH) due to their severe course and unfavorable prognosis, which is considerably associated with the insufficient effectiveness of standard treatment approaches^2,3^. The functional state of the right ventricle is the main limiting factor determining the prognosis of patients with pulmonary hypertension. Moreover, it depends primarily on the structural changes of the pulmonary circulation vessels. Thickening and irreversible deposition of collagen in the walls of the pulmonary artery branches leads to rapid disease progression and a decrease of therapy effectiveness. In this regard, histological examination of the pulmonary circulation vessels is one of the crucial values both in preclinical studies, when studying the pathogenesis of the disease and testing new therapeutic approaches, and in clinical practice when studying surgical material. At the same time, calculating the hypertrophy index of vascular wall in all branches of the pulmonary artery identified on the section is one of the most accurate and valid methods for assessing remodeling of the pulmonary circulation vessels^4,5^. The advantages of this method are: integrity of the obtained data; a large amount of information for analysis; possibility of blinded data analysis; possibility of subsequent analysis of vessels subgroups (for example, depending on the external diameter of the vessel). However, manual evaluation of the hypertrophy index and the outer diameter of the vessels is complicated by a significant investment of time and the requirement for training a specialist to analyze micrographs. Thus, according to the data of the performed work, an experienced researcher spends approximately 4 minutes to measure the parameters of a vessel. The presented database includes 609 vessels, therefore, for analysis, one highly qualified researcher has to take out approximately 41 hours of work, which makes it difficult for wide implementation.

The number of vessel segmentation methods at the junction of medical imaging and computer vision is growing. Despite this, the topic of histology segmentation is less popular. The publications mention several datasets for vessel segmentation. The PAIN (Pathology Artificial Intelligence Platform) dataset^6^ contained 100 whole-slide histopathology images, 60 of them have two-layers of annotation for the viable tumor area and the whole tumor area, the BACH (BreAst Cancer Histology)^7^ aimed at the classification and localization of clinically relevant histopathological classes in microscopy. It is composed of 400 training and 100 test images, with the four classes. In^8^ authors collected 399 whole-slide images and corresponding glass slides of sentinel axillary lymph nodes. Data was got from 399 patients that underwent surgery for breast cancer.

Recent papers propose a deep learning-based approach for vessel segmentation with a U-Net backbone, and an EfficientNet encoder in^9^ or multi-scale multi-encoder models in^10^ and in^11^ based on FCN architecture. The authors of^12^ also offer their own framework for automatic nuclear instance segmentation and classification and proposed the network.

## Methods

**Fig. 1:**
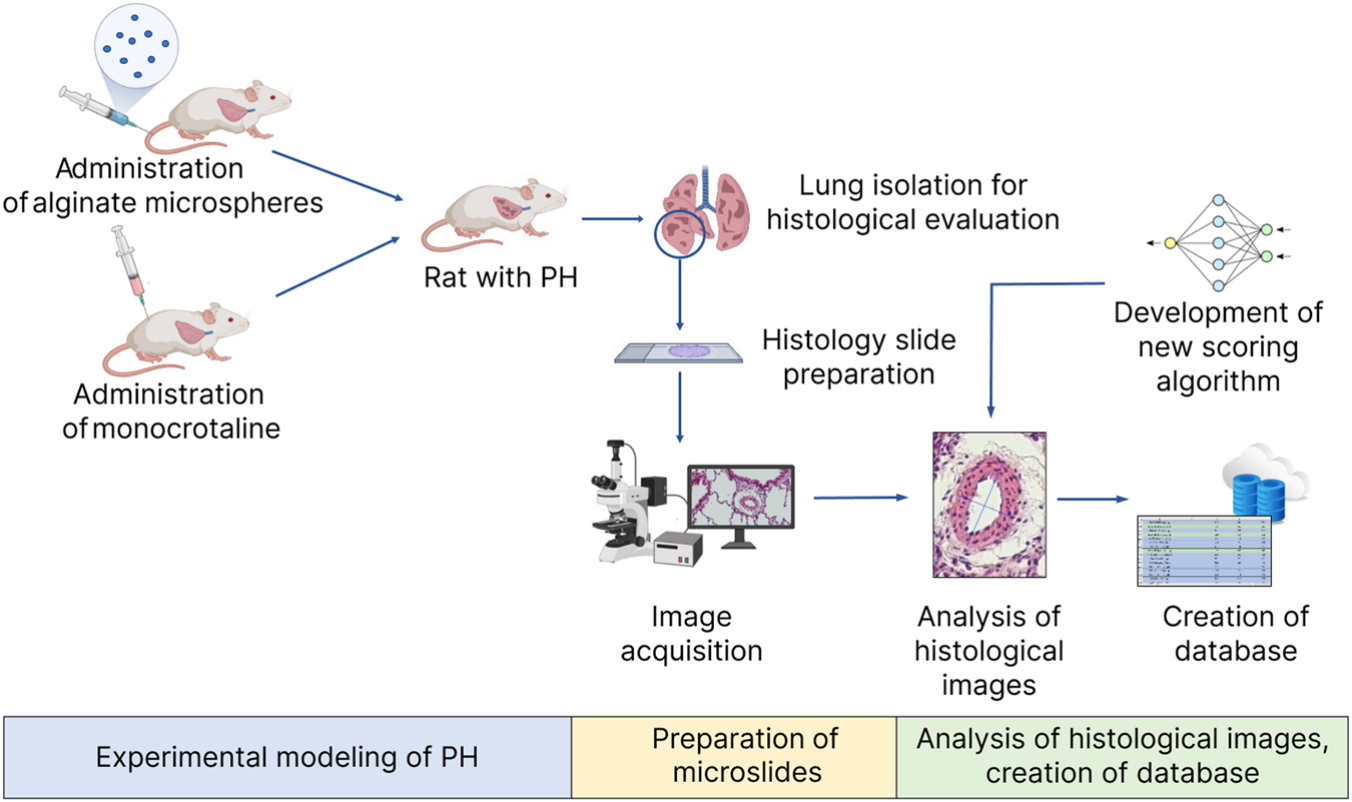
Study design. The study design includes three stages: 1. Experimental modeling of two types of PH: CTEPH and monocrotaline-induced PH. 2. Preparation of microslides and image acquisition. 3. Analysis of histological images, creation of database using development of new scoring algorithm.

The experimental design is summarized in Fig. 1. The database of histological micrographs is based on a series of preclinical studies, both previously conducted^5,13,14^ and those that have not yet been published. In all studies, male Wistar rats were used, total 244 animals. For CTEPH modeling, repeated embolization of the vascular bed with partially biodegradable microspheres was used^14^. Microspheres were prepared from ultrapure sodium alginate (Sigma-Aldrich, USA) using an electrostatic encapsulator (B-390, Switzerland). A 2% barium chloride solution was used as a stabilizing agent. 50 μl of microspheres were suspended in 1 ml of saline and were injected into the tail vein 8 times at 4 days interval. Modeling of iPAH was performed using a single subcutaneous injection of monocrotaline (Sigma-Aldrich, USA) at a dose of 60 mg per kg of body weight^15^. Histological examination of the lungs was carried out at different periods after modeling pulmonary hypertension (from 1 to 19 weeks), depending on the purposes of the study. The simulated pathologies are reproduced with high accuracy by the use of these models. Thus, it was previously demonstrated that use of partially biodegradable sodium alginate microspheres with repeated intravenous administration is characterized by a stable increasing in the right ventricle systolic pressure, residual obstruction of the vascular bed, a decreasing in exercise tolerance, as well as severe remodeling of the branches of the pulmonary artery^13–15^. All of these changes characterize CTEPH^16^. The monocrotaline-induced pulmonary hypertension model is the most popular and representative experimental model of pulmonary arterial hypertension. The effectiveness of this model has been demonstrated in many previous studies^17,18^. The right lower lobe of the lung was isolated for histological evaluation; it was divided into 4 transverse levels for analysis. The selection of the lung lobe is based on the optimal shape and size for histological examination. The data obtained from the analysis of this lobe are representative for the whole lung. Sections 3 × 5 *μm* thickness were stained with hematoxylin and eosin. The preparation was carried out using an Eclipse Ni-U microscope (Nikon, Tokyo, Japan) with a magnification from × 5 to × 40. Microscopic results were evaluated using Nis Elements Br4 software (Nikon, Tokyo, Japan) and ImageJ 1.53k (Wayne Rasband (NIH), USA). In all identified vessels belonging to the branches of the pulmonary artery, the average outer diameter of the vessel and the hypertrophy index, which is the ratio of the vascular wall area to the entire vessel area in percent, were determined on two distal sections of the lung. The total number of analyzed vessels was **609**.

On the image pre-processing stage, masking should be implemented for the dataset. Masking is an image processing technique in which a tiny ‘image piece’ is defined and used to alter a bigger picture. Many forms of image processing, such as edge detection, motion detection, and noise reduction, are based on the masking process. Convoluting a mask with an image is another term for filtering. Filter masks are often known as convolution masks because the procedure is similar to convolution. So, before training the model, make masks for all images in the dataset.

## Data Records

**Fig. 2:**
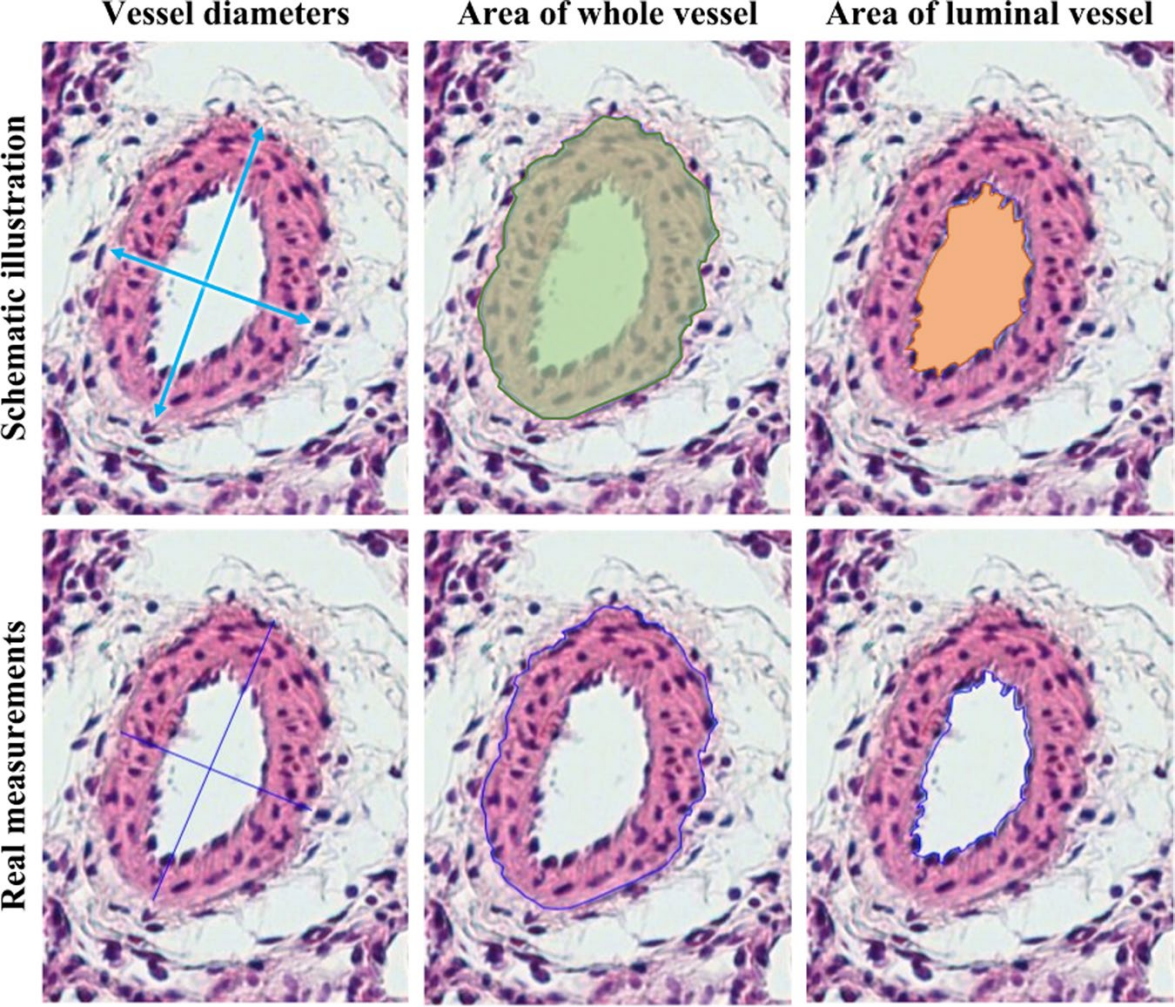
.Histological measurements of vessels. Schematic illustration of measurements at the top row and real measurements in ImageJ at the bottom.

**Fig. 3:**
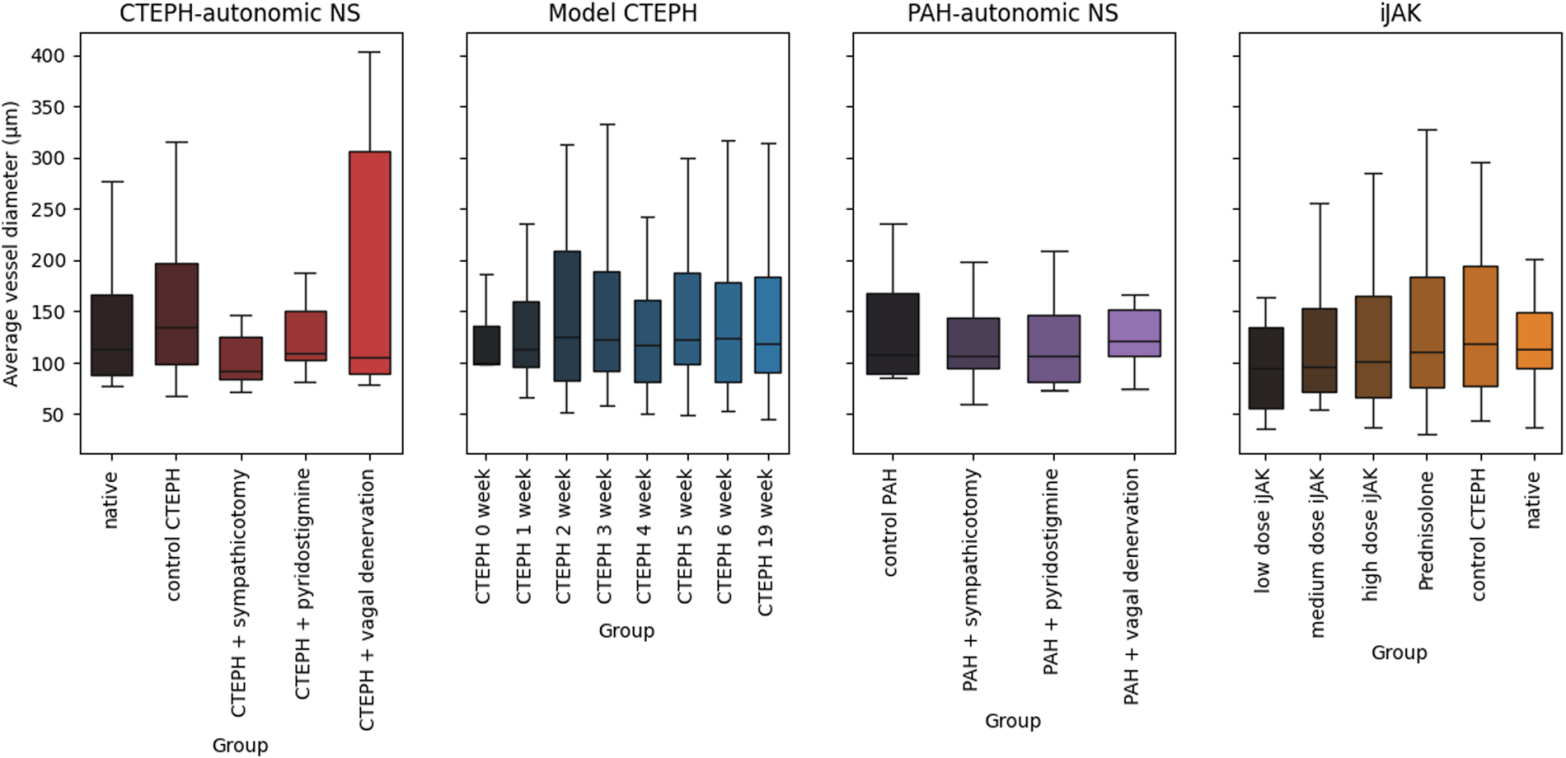
Boxplots for average vessel diameter (*μm*). Each panel represents a single project. Each item represents a distribution of an average vessel diameter in subgroup of a related project.

**Fig. 4:**
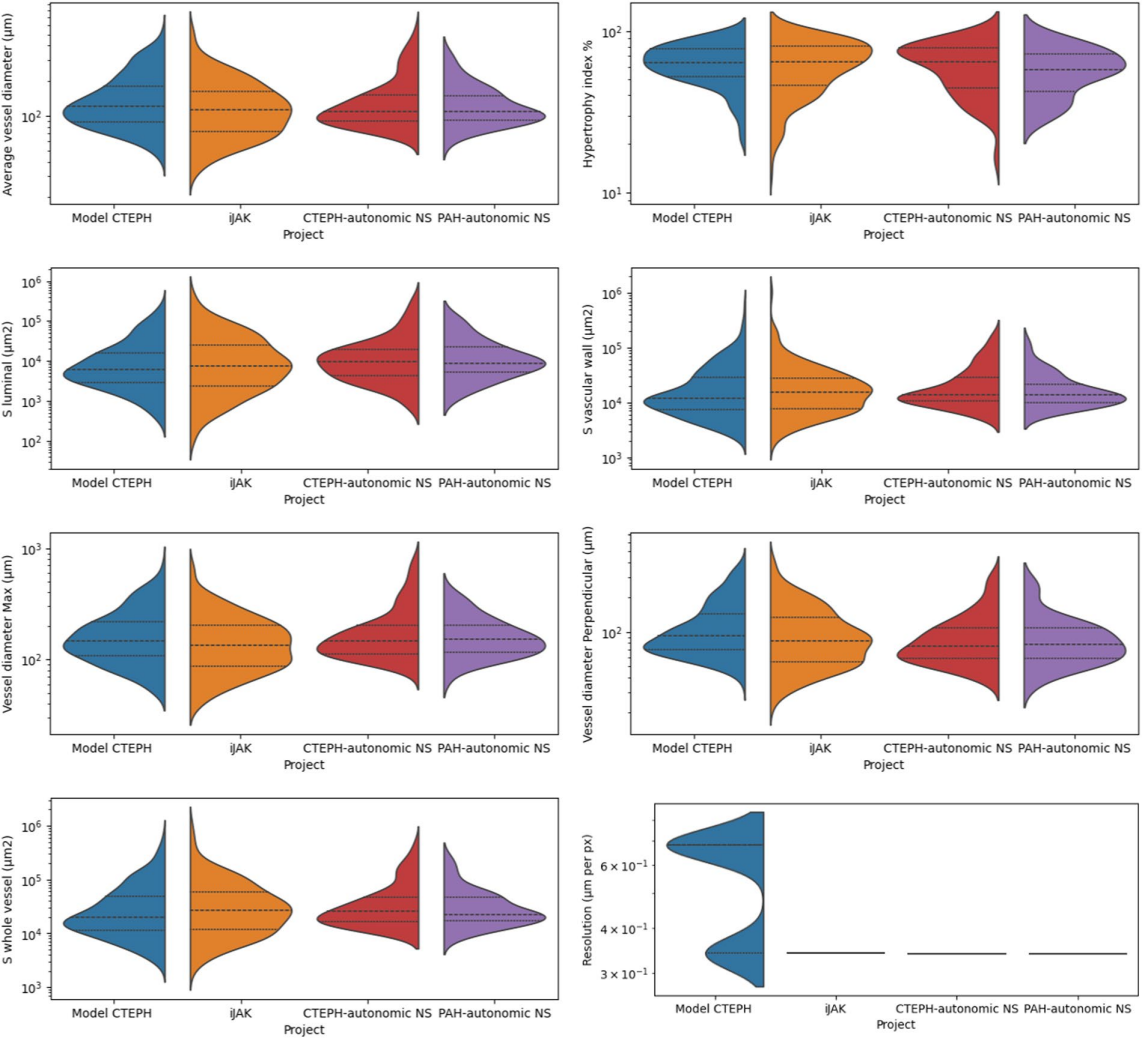
Distribution of features across projects. Dotted lines show the quartiles of the data distributions.

The data record contains a set with **609** vessels images, which were collected during 4 studies. Each image from the dataset is annotated with the following information (see Figs. 2, 3 and 4). Data sets acquired during the project except Model CTEPH, the **image resolution** is equal to 0.34 *μm*/*px*. For Model CTEPH the additional resolution is 0.68 *μm*/*px*.

1. Vessel diameter Max, *μ*m (*D*_*max*_[*μm*]).
2. Vessel diameter Perpendicular, *μ*m (*D*_*p*_[*μm*]).
3. Average vessel diameter, *μ*m (*D*_*mean*_[*μm*]).
4. Square of the whole vessel, *μ*m^2^ (*S*_*v*_[*μm*^2^]).
5. Square of the luminal, *μ*m^2^ (*S*_*l*_[*μm*^2^]).
6. Square of the vascular wall, *μ*m (*S*_*w*_[*μm*^2^]).
7. Hypertrophy index, % (*H*).

**Fig. 5:**
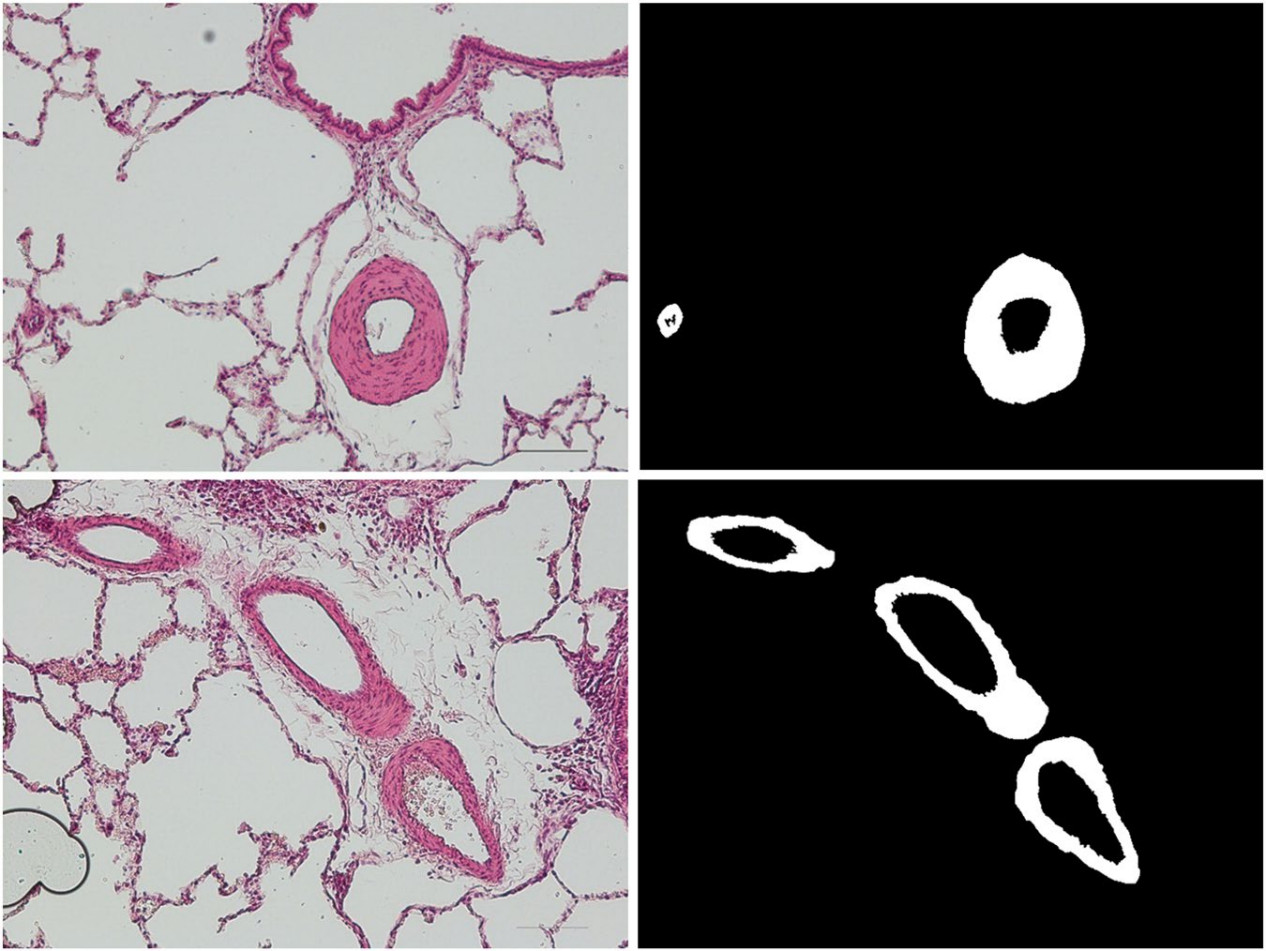
Left column is an original cross-sectional microscopic samples. In the right column is presented a relevant masks.

Based on the contours highlighted by experts (as mentioned in section), binary masks were prepared. In Fig. 5 samples from the dataset are presented, along with their masks.

The dataset is organised as following:

- BASE_20240410.xlsx – the table with metadata of images.
- images.zip – archive with microscopic PNG images.
- masks.zip – archive with masks for microscopic images in PNG format.
- Images with outline.zip – archive with microscopic images with contours highlighted by experts.

## Technical Validation

The numerical characteristics have been automatically validated with the criteria described in the Table 1. These criteria have been selected according to the following justification.

**Table 1:** Technical Validation criteria.

Parameters	Minimal	Maximum
Vessel diameter Max, *μm*	28	3440
Vessel diameter Perpendicular, *μm*	20	822
Average vessel diameter, *μm*	24	1830
S whole vessel, *μm*^2^	447	1426036
S luminal, *μm*^2^	57	581758
S vascular wall, *μm*^2^	390	1423009
Hypertrophy index, %	13	100

### Vessel diameters min and max verification

The following database does not include capillaries, so the diameters of the pending vessels should exceed the size of the lung capillaries. The standard capillary diameter is 5-10 *μ*m^19,20^, therefore the minimum values of the average and maximum vessel diameters should not be less than 11 *μ*m. The perpendicular diameter, taking into account the possibility of the vessel wall deformation, should not be less than the arteriole wall thickness x 2 microns.

The average diameter of the rat pulmonary trunk normally ranges from 1600 to 2000 *μm*^21,22^. However, against the background of pulmonary hypertension, it could expand significantly. Therefore, the maximum value of vessel diameters was taken to be three times higher than the normal values (6000 *μm*). The maximum diameters of the vessels are specified in (1).

1 $$11[\mu m]\leqslant {D}_{max}\leqslant 6000[\mu m],\quad 11[\mu m]\leqslant {D}_{mean}\leqslant 6000[\mu m],\quad 2[\mu m]\leqslant {D}_{p}\leqslant 6000[\mu m]$$

In the available literature there are no indications of the normal areas of pulmonary vessels in rats. Therefore, the normal range for areas was calculated from the previously specified linear dimensions of the vessels.

### S whole vessel verification

According to equation (2) S whole vessel range should be in limits 95 - 28260000 *μm*^2^ due to the min and max criteria for average diameters (11-6000 *μ*m).

2 $$95[\mu {m}^{2}]\leqslant {S}_{v}\leqslant 28260000[\mu {m}^{2}]$$

### S vascular wall verification

The minimum S vascular wall value is 75 *μm*^2^ based on the arteriole wall thickness with a minimum diameter (vessel diameter - 11 *μ*m, wall thickness - 2.5 *μ*m). The maximum value should not exceed S whole vessel by (3).

3 $$75[\mu {m}^{2}]\leqslant {S}_{w}\leqslant {S}_{v}$$

### S luminal verification

Minimum value could be 0 *μm*^2^ according to obliteration (complete closure) of the vessel lumen against its structural changes. According to equation (4) the maximum value should not exceed S whole vessel.

4 $$0[\mu {m}^{2}]\leqslant {S}_{l}\leqslant {S}_{v}$$

### Hypertrophy index

The hypertrophy index could be 100% in case of complete obliteration of the vessel lumen. The minimum hypertrophy index tends to zero (based on the normal range for S luminal and S whole vessel) and could not be negative according to equation (6).

5 $$H=\frac{{S}_{v}[\mu {m}^{2}]-{S}_{l}[\mu {m}^{2}]}{{S}_{v}[\mu {m}^{2}]}\times 100 \% $$

6 $$0[ \% ]\leqslant H\leqslant 100[ \% ]$$

## Usage Notes

To demonstrate the possibility of using a segmentation-related part of the dataset, a pipeline with an example of neural network training and use is provided as part of the dataset in the repository^23^.

## Data Availability

The source code of the baseline, as well as a direct link to the Pulmonary Circulation Vessels Dataset, is available in the GitLab repository^23^. The installation of Python and Jupyter using the virtual environment is recommended, with the necessary technical instruction supplied in the “ReadMe.md” inside the repository. The dataset is publicity available at figshare^24^.
